# Evaluation of the Structural Validity of the Work Instability Scale Using the Rasch Model

**DOI:** 10.1016/j.arrct.2021.100103

**Published:** 2021-01-13

**Authors:** Ze Lu, Joshua I. Vincent, Joy C. MacDermid

**Affiliations:** a School of Rehabilitation Science, McMaster University, Hamilton, Ontario, Canada; b Roth McFarlane Hand and Upper Limb Centre, St. Joseph’s Hospital, London, Ontario, Canada; c School of Physical Therapy, Western University, London, Ontario, Canada

**Keywords:** Arthritis, rheumatoid, Occupational health, Presenteeism, Rehabilitation, Work, Work performance, DIF, differential item functioning, ICC, item characteristic curve, LD, local dependency, OA, osteoarthritis, PCA, principal component analysis, PSI, person separation index, RA, rheumatoid arthritis, WD, work disability, WI, work instability, WIS-23, Work Instability Scale 23-item version, WIS-WRUED, Work Instability Scale for work-related upper extremity disorders

## Abstract

**Objective:**

To use Rasch analysis to examine the measurement properties of the 23-item version of the Work Instability Scale (WIS-23) in a sample of worker compensation claimants with upper extremity disorders.

**Design:**

Secondary data analysis on the data retrieved from a cross-sectional study.

**Setting:**

Tertiary care hospital.

**Participants:**

Patients (N=392) attending a specialty clinic for workers with upper limb injuries at a tertiary hospital were prospectively enrolled.

**Interventions:**

Not applicable.

**Main Outcome Measures:**

WIS-23.

**Results:**

The study sample contained 392 participants between the ages of 19 and 73 years (mean, 47.0±10.5y). There were 148 (37.8%) women, 182 (46.4%) men, and 62 (15.8%) participants for whom sex identification was unavailable. The initial WIS data analysis showed significant misfit from the Rasch model (item-trait interaction: χ^2^=293.52; *P*<.0001). Item removal and splitting were performed to improve the model fit, resulting in a 20-item scale that met all assumptions (χ^2^=160.42; *P*=.008), including unidimensionality, local independence of items, and the absence of differential item function based on age, sex of respondents, employment type, and affected upper extremity area across all tested factors.

**Conclusion:**

With the application of Rasch analysis, we refined the WIS-23 to produce a 20-item WIS for work-related upper extremity disorders (WIS-WREUD). The 20-item WIS-WREUD demonstrated excellent item and person fit, unidimensionality, acceptable person separation index, and local independency. The WIS-20 may provide better measurement properties, although longitudinal psychometric evaluations are needed.

The assessment of health-related at-work limitations is essential for clinical researchers to evaluate the effect of occupational injuries.^1^, ^2^, ^3^ Previous research suggests that productivity loss at work contributes to a considerable amount of indirect economic costs.^4^ Upper extremity injuries are commonly considered as the major source of work disability.^5^^,^^6^ Some clients have complicated career trajectories that include career changes and job adjustments that may relate to a mismatch between person’s functional capabilities and the demands of the job. This is defined as work instability (WI).^7^, ^8^, ^9^ WI can be related to a variety of factors, including decreased capability related to aging or disease or increased capability due to treatment effects. Work instability can also occur when the demands of the job change.

The WI Scale (WIS-23) was originally developed as a WI classification system for individuals with rheumatoid arthritis (RA) to support timely and appropriate management, including vocational rehabilitation, psychosocial support, and clinical intervention for job retention.^7^^,^^10^ It consists of 23 dichotomous items (yes or no) describing specific experiences or situations that provide indications of WI. Examples of individual items include: “I have pain or stiffness all the time at work.” The total score is a sum between 0 and 23, with higher scores indicating greater WI. Three levels are used to classify the total score into low WI (<10), indicating low risk of work disability (WD); moderate WI (10-17), corresponding to medium level of WD; and high WI (>17), indicating those individuals having high risk of WD.^7^ Workplace modification would be recommended for individuals (4 of 5) with a moderate level of WI and is critical for those (19 of 20) who have a high level of WI.^7^

Previous studies have affirmed acceptable reliability and construct validity under classical test theory in populations with RA and osteoarthritis (OA).^7^^,^^11^ In addition, a qualitative interview captured key themes of WIS-23 covering job flexibility, good working relationships, and symptom control.^11^ Several formats of the WIS have been developed based on different diagnoses and populations, including upper extremity disorders,^12^ manual workers,^13^ brain injuries,^14^ and OA.^10^

A large study involving 2092 participants concluded that individuals with work-related injuries had a higher risk of absence from work, mobility-related functional problems, disability, and impaired functioning related to anxiety or depression.^15^ Workers who experience musculoskeletal pain across various regions will seek diverse resources of health care, in comparison to those with an explicit health condition.^13^ This suggests the need for a universal version of WIS for work-related upper extremity disorders (WIS-WRUED).

Rasch analysis, is an alternative strategy for the evaluation of structural validity. Rasch analysis enables examination of the assumption of unidimensionality and structure of rating scales to convert the ordinal scale of individual items into interval scaling.^16^^,^^17^ To compute a total score, the response options should demonstrate interval level scaling, also known as the scaling assumption.^17^ Where outcome measures are not developed using Rasch modeling, they can be retrospectively evaluated for fit to the Rasch model, which often results in modifications to the questionnaire to obtain fit. Previous studies evaluated the WIS-23 using Rasch analysis and found a significant deviation from the Rasch model fit.^10^^,^^12^

Therefore, the purpose of this study was to apply Rasch analysis to (1) examine to what extent the rating scale of WIS-23 fits to the Rasch model by inspecting the test fit statistic and ordering of item thresholds; (2) examine the differential item functioning based on age, sex of respondents, employment type, and affected upper extremity area and exploring solutions by altering the rating scale; and (3) test the construct validity of all subscales of the Work Limitations Questionnaire by examining the unidimensionality and local dependency.

## Methods

The study dataset was prospectively collected from a specialty clinic for workers with upper limb injuries in a tertiary hospital in Canada. The package of patient-reported outcome measures including WIS-23 was sent to patients shortly before their initial clinic assessment and completed immediately prior to attending clinic or at the initial clinic visit. Research ethics board approval was obtained for the clinical database, and patients provided informed written consent to have data used in research.

Rasch analysis included tests of unidimensionality fit of residual, ordering of item thresholds, person separation index (PSI), differential item functioning (DIF), and local independence of items. The analysis was performed using RUMM 2030 professional suite software.a The significance level was set at 0.05, with Bonferroni correction applied when multiple comparisons were made. To facilitate stable analyses, sample sizes of 250 participants were required.^18^^,^^19^

### Rasch analysis

#### Test of fit

The test of fit quantifies to what extent items from the outcome measure meet the expectations of the Rasch model. Fit statistics can be checked at both overall and individual item levels. For the overall fit, the *P* value from a chi-square test of item-trait interaction should be nonsignificant according to the critical value.^17^^,^^20^ The item–person interaction statistics were then transformed to approximate a *z* score following a standardized normal distribution. We expected a mean of approximately 0 and a standard deviation of 1 to characterize the normal distribution.^17^ At the individual level, a fit residual localized within ±2.5 logits represented an adequate fit for the model.^17^

#### Threshold

The threshold refers to the point between 2 response categories at which either response is equally probable. Disordered thresholds mean that the respondents fail to meaningfully discriminate between response options or that options are potentially confusing. Threshold maps and categorical probability curves were used for visual inspection of this phenomenon. Where needed, we attempted to resolve this problem by collapsing adjacent categories and reversing the order of response options.^17^

#### Targeting

Scale-to-sample targeting reflects the extent to which the items can measure the whole range of an individual’s ability level. The person item threshold distribution displays the relative difficulty (item locations) and relative ability (person location) on the same ruler of logits. The better the ranges match each other, the greater the potential for precise person measurement. Poor targeting often results in floor or ceiling effects, indicating that patients at the extremes cannot be differentiated from each other nor can change be measured in terms of lower (floor) or higher (ceiling) future scores.^20^^,^^21^ A scale is considered well targeted if the difference between person and item means would be less than 1 logit unit.^10^

#### DIF

DIF occurs when individual groups of patients within the study sample (men vs women), respond differently to an item given the equal level of characteristic being measured. For instance, men and women with equal level of work disability may respond systematically different to an item measuring completeness of job demands.^22^ Two types of DIF can be identified. Uniform DIF is where the group shows a consistent systematic difference in their responses to an item. The standard approach of splitting the item for individual groups can be used to address such issue. Nonuniform DIF results from random differences (eg, responses to individual item vary across levels of the ability for subgroups). Currently, there is no solution for nonuniform DIF, except item removal.^23^ DIF was examined on age, sex of respondents, employment type, and affected upper extremity area using both critical statistical values using a Bonferroni adjusted *P* value of .0007 (.05/23∗3) and visual inspection by item characteristic curve (ICC).^17^ The visual inspection was facilitated by plotting the item characteristic curve along with the person trait for given person factors. Under an ideal situation, there is no difference in ICCs for different groups, indicating that participants with identical level of ability have equal probabilities of affirming a given item.

#### Dimensionality

The basic assumption of the Rasch model, unidimensionality, was checked through the principal component analysis (PCA) under item response theory. After the rescoring of individual response options and any resultant item reduction, the PCA was be revisited as confirmation of the unidimensionality.^24^ We set the number of significant *t* tests at 5% of the total comparisons as the indicator of multidimensionality.

#### Local independence

Residual pattern refers to the standardized person-item differences between the observed data and expected response generated by the model for every person’s response to individual item. The Rasch model requires no residual pattern existing in the data, which is named as local independence. Such residual pattern was examined by PCA. A residual correlation between any 2 items greater than 0.2 above the average correlation would appear to indicate the violation of local independence, as local dependency (LD).^25^^,^^26^ Potential reasons for the appearance of LD are response dependency and multidimensionality.^27^ Item deletion and the creation of testlets to bundle the dependent items was used as a potential solution to address the LD.^25^^,^^28^

#### Reliability

The PSI indicates the precision of the estimate for each person and was used to evaluate the internal consistency under the Rasch model. The acceptable value was set as 0.7 to establish that the scale is reliable to distinguish between at least 2 groups.^29^, ^30^, ^31^ In addition to the PSI, the traditional Cronbach’s alpha was provided by the RUMM 2030 program; 0.8 was adopted as the satisfactory value.^32^

## Results

### Study participants

The study sample contained 392 participants with full responses. The age of participants ranged from 19 to 73 years old (mean ± SD, 47.0±10.5y). There were 148 (37.8%) women, 182 (46.4%) men, and 62 (15.8%) participants missing sex information. Within the study sample, 56.4% of the total subjects engaged in labor work, 16.8% engaged in office work, and 7.9% engaged in a job that included both labor and office work. Due to the nature of Rasch analysis, continuous descriptive variables were transferred to categorical data. The continuous age variable was then recoded into 2 groups according to the median value. Specifically, code 1 represented the group between 19 and 49 years old, and code 2 represented those between 50 and 73 years old. A full summary of participant demographic and clinical characteristics is listed in table 1.

**Table 1:** Demographics of the total sample (N=392)

Personal Factor	Classification	Frequency	Percentage
Sex	Women	148	37.8
	Men	182	46.4
	Missing	62	15.8
Affected side	Left	119	30.4
	Right	177	45.2
	Both	76	19.4
	Missing	20	5.0
			
Job	Labor	221	56.4
	Office	66	16.8
	Mixed	31	7.9
	Missing	74	18.9
			
Injury region	Wrist and hand	99	25.3
	Elbow and forearm	57	14.5
	Shoulder and arm	171	43.6
	Upper extremity (CRPS)	12	3.1
	Other	6	1.5
	Missing	47	12.0
			
Age, y	Mean ± SD	Median	Range
	47.0±10.5	49	19-73
Abbreviation: CRPS, complex regional pain syndrome.			

### Test of fit

The rating scale model was selected for the current analysis due to the unified dichotomous response options over all 23 times.^27^ The initial evaluation of the overall questionnaire with 23 items demonstrated poor overall fit to the Rasch model in the substantial deviation in the standardized item fit residual statistic (mean ± SD, –0.27±1.9). The significant chi-square test (χ^2^=293.52; *df*=138; *P*<.001) for item-trait interaction revealed misfit from Rasch model. Table 2 lists the overall summary of fit statistics. To locate problematic items that may cause misfit issues, we checked the individual item fit statistics. Items 1 and 23 were misfitting as the fit residual values were greater than 2.5. Individual item descriptions with Rasch solutions are shown in table 3.

**Table 2:** Overall summary of Rasch statistics

Analysis	Sample Size		Item Fit Residual	Person Fit Residual	Chi-Square Interaction			PSI	Alpha	Unidimensionality *T* Tests		
	With ext	Without ext	Mean ± SD	Mean ± SD	Value	*df*	*P* Value			No. of significant tests	N	%
Original 23-item	392	384	–0.27 ± 1.9	–0.23 ± 0.78	293.52	138	<.0001	0.85	0.87	19	384	4.95
Revised 20-item	392	381	–0.38 ± 1.25	–0.25 ± 0.73	160.42	120	.008	0.85	0.87	12	381	3.15

Abbreviation: ext: extreme data. NOTE. The original work instability index contains 23 items. To achieve the Rasch model fit, 4 items, including items 1 and 23, were removed. Item 19 was deleted due to the issue of nonuniform DIF in the job classification. Item 11 was separated for men and women, and items 18 and 22 were separated for different affected regions due to the presence of uniform DIF. No disordered thresholds were found in the original or revised versions. After Bonferroni correction, the significant *P* value was .0027.

**Table 3:** Individual item description with Rasch solutions

	Item Description	Solution	Rationale
1	I can get my job done, I’m just a lot slower	Remove	Fit residual 5.92
2	If I don't reduce my hours, I may have to give up work		
3	I am very worried about my ability to keep working		
4	I have pain or stiffness all the time at work		
5	I don't have the stamina to work like I used to		
6	I have used my holiday so that I don't have to go on sick leave		
7	I push myself to go to work because I don't want to give in to my shoulder/elbow problem		
8	Sometimes I can't face being at work all day		
9	I have to say no to certain things at work		
10	I've got to watch how much I do certain things at work		
11	I have great difficulty opening some of the doors at work	Item split	Uniform DIF identified in sex
12	I have to allow myself extra time to do some jobs		
13	It's very frustrating because I can't always do things at work		
14	I feel I may have to give up work		
15	I get on with work but afterwards I have a lot of pain		
16	When I’m feeling tired all the time, work's a grind		
17	I'd like another job, but I am restricted as to what I can do		
18	I'm getting up earlier because of my shoulder/elbow problem	Item split	Uniform DIF identified in injury region
19	I get very stiff at work	Remove	Nonuniform DIF identified in job
20	I'm finding my job is about all I can manage		
21	The stress of my job makes my shoulder/elbow problem flare		
22	I'm finding any pressure on my hands is a problem	Item split	Uniform DIF identified in injury region
23	I get good days and bad days at work	Remove	Fit residual 2.86

### Threshold

**Fig 1:**
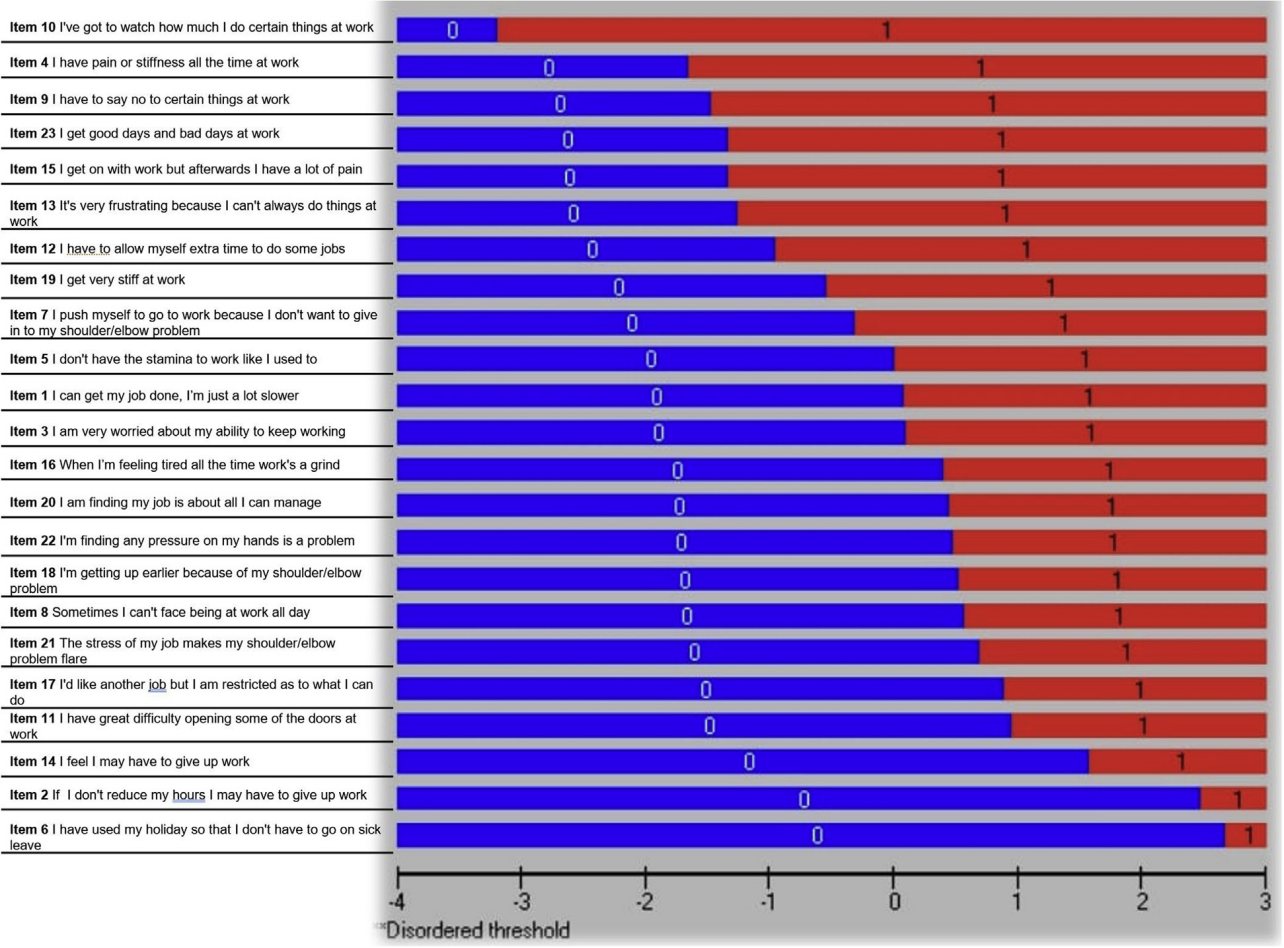
The original thresholds map for the WIS-23. All thresholds are ordered and displayed on the map.

The inspection of the threshold map of WIS-23 suggested that no disordered issues were present, as expected given the dichotomous response option. Figure 1 shows the threshold map of the original WIS.

### Targeting

**Fig 2:**
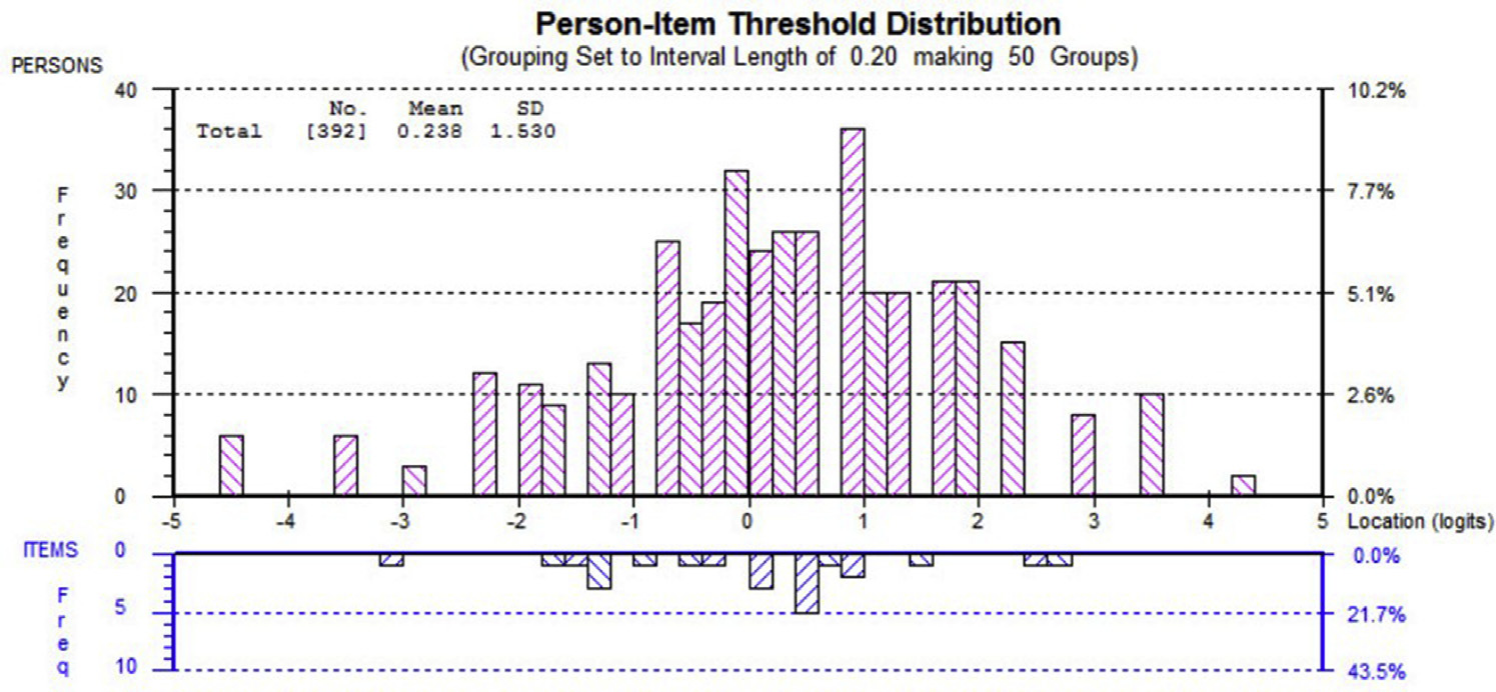
Person-item distribution map for the WIS-23. The mean of person logits is equal to 0.24 (<1 logit), indicating that the scale item difficulty matches the abilities of the sample. In addition, the floor and ceiling effects were not detected as only 6% (<10%) of the participants were on either end of the spectrum.

Figure 2 shows the targeting of the WIS-23. The mean of person logits is equal to 0.24 (<1 logit), indicating that the scale item difficulty matches the abilities of the sample. In addition, the floor and ceiling effects were not detected because only 6% (<10 %) of the participants were on either end of the spectrum.

### DIF

**Fig 3:**
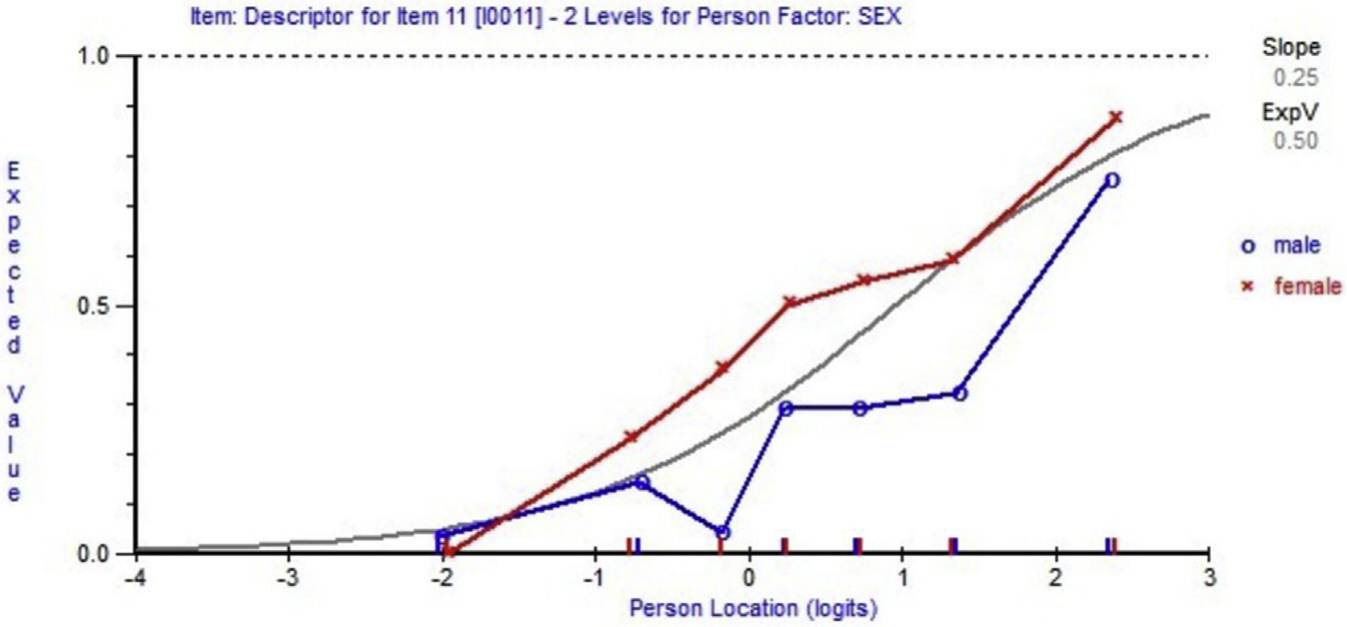
Uniform DIF detected on item 11 across male vs female groups. The visual inspection was facilitated by plotting the item characteristic curve along with the person trait for given person factors.

The personal factors considered as potential sources of DIF (bias) were sex, age (19-49y and 50-73y), affected sides (left, right, bilateral), injured body area (wrist and hand, elbow and forearm, shoulder and arm, entire upper extremity), and 3 job categories (labor, office, and mixed). Uniform DIF due to sex bias was detected on item 11, as the curve representing expected value of item response for women was higher than the curve for men across most person locations (person ability). Figure 3 shows the uniform-DIF of item 11. Similar uniform DIFs were identified on items 18 and 22 for injured body area due to selection bias. Item 19 was removed due to the nonuniform DIF identified in different job categories. Table 3 lists the individual item description with Rasch solutions.

### Dimensionality

The WIS-23 met the assumption of unidimensionality as 4.95% (<5%) of the independent *t* tests were found to be significant at the 5% level (see table 2 for the overall summary of fit statistics).

### Local independence

None of the item-to-item correlations exceeded the cutoff value, and the assumption of local independence was met in the WIS-23 (Supplemental Table S1, available online only at http://www.archives-pmr.org/).

### Reliability

The PSI value was equal to 0.85 for the WIS-23. The Cronbach’s alpha was calculated as 0.87 without missing data points.

### Revisiting the Rasch statistics

**Fig 4:**
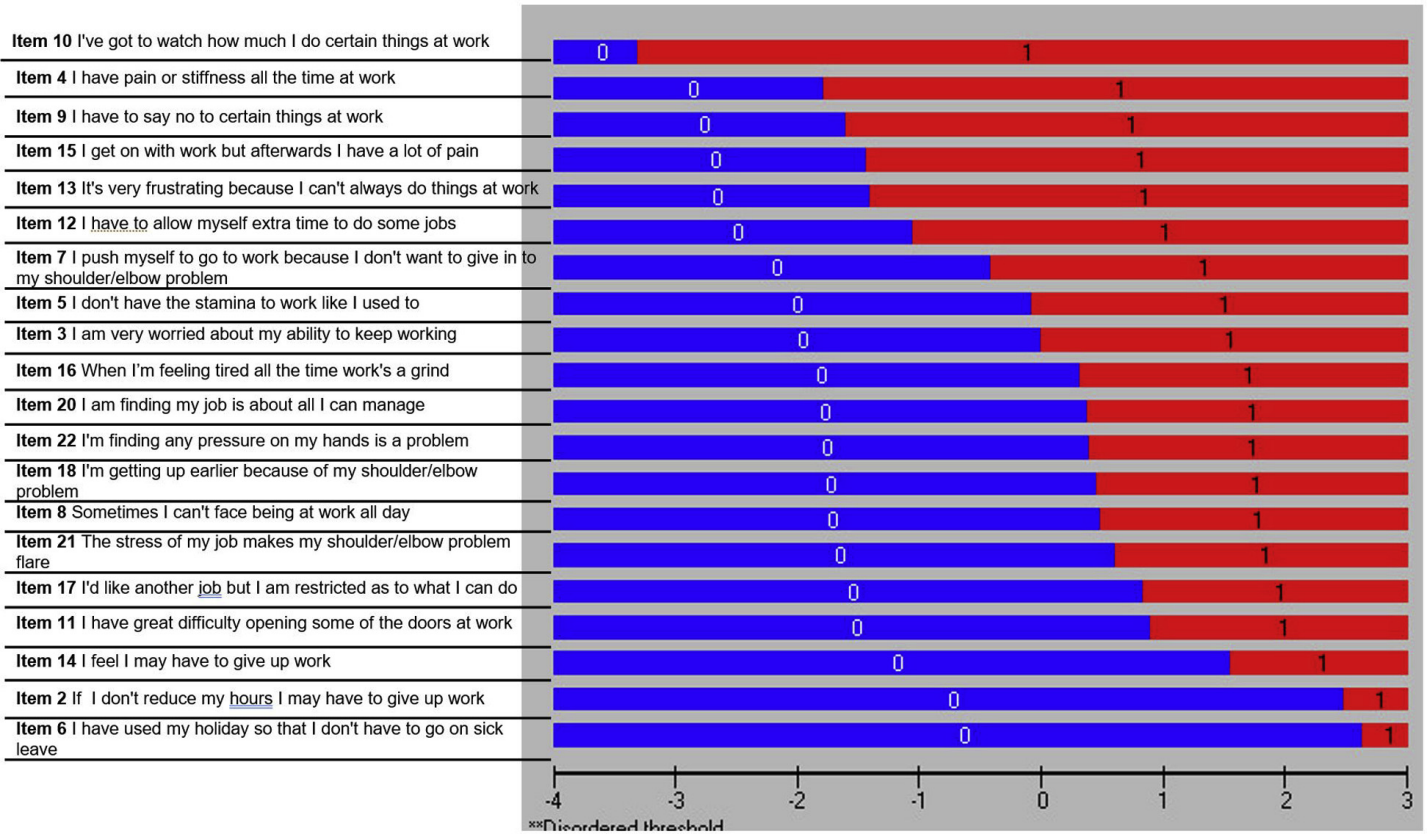
The reassessment of the thresholds shown by the ordered threshold map for the revised WIS. All thresholds are ordered and displayed on the map.

**Fig 5:**
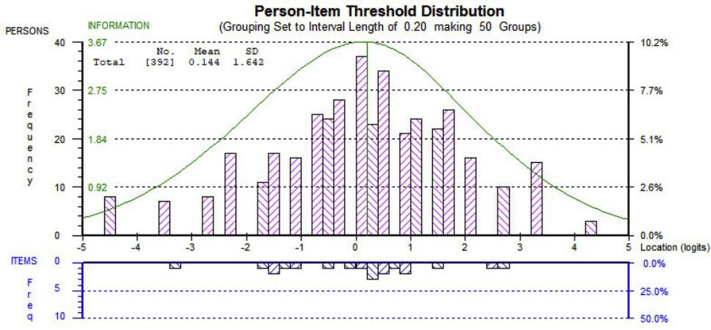
Person-item distribution map for the revised 20-item version of the WIS. The mean of person logits is equal to 0.14 (<1 logit), indicating that the scale item difficulty matches the abilities of the sample. In addition, the floor and ceiling effects were not detected as only 8% (<10%) of the participants were on either end of the spectrum.

After removal of 3 items with unresolvable issues, including item misfit (items 1 and 23) and nonuniform DIF (item 19), the revised WIS questionnaire contained 20 items. The reanalyzed WIS-20 showed acceptable level of overall fit to the Rasch model (mean ± SD, –0.38±1.25) with a nonsignificant chi-square test (χ^2^=160.42; *df*=120; *P*=.008) for item-trait interaction (see table 2). No disordered thresholds were revealed during the reanalysis (fig 4) A mean value of 0.14 logits (<1 logit) revealed good targeting of the revised WIS. Flooring and ceiling effects were absent in the 20-item version (fig 5). The revised version was in accordance with the assumptions of dimensionality and local independency. Both PSI and reliability statistics remained the same after deleting 3 items (see table 2). Therefore, we conducted a logit transformation of the 20-item WIS summed scores to provide interval-level scaling. The conversions are presented in table 4.

**Table 4:** Transformation of WRUED-WIS raw scores to interval-level scores on a logit scale summed

Summed Score	Logit	Interval
0	–4.493	0.0
1	–3.47	2.3
2	–2.736	4.0
3	–2.21	5.2
4	–1.785	6.2
5	–1.42	7.0
6	–1.091	7.7
7	–0.788	8.4
8	–0.503	9.1
9	–0.23	9.7
10	0.035	10.3
11	0.299	10.9
12	0.564	11.5
13	0.838	12.1
14	1.126	12.8
15	1.438	13.5
16	1.787	14.3
17	2.192	15.2
18	2.691	16.3
19	3.372	17.9
20	4.303	20.0

## Discussion

Our Rasch analysis of WIS-23 supports a 20-item version and provides a transformation of interval level scaling in injured workers with upper extremity conditions. This complements the findings from a previous study by Tang et al,^12^ in which they recommended a 17-item WIS specifically for the upper extremity. They excluded items 1 and 23 among others, which were also recommended to be removed by the current Rasch analysis.

Our initial analysis indicated that there were no disordered thresholds. This was an expected finding as dichotomous response options are not susceptible to disordered thresholds, as reported in a previous Rasch analysis study.^12^

PSI values of >0.70 and >0.85 are considered acceptable for group and individual use, respectively. The PSI value for the 20-item WIS was 0.85, indicating that the WIS can discriminate at both levels and show similar discrimination as the WIS-23 (PSI=0.83).^12^

We had to delete 2 misfitting items, including items 1 (“I can get my job done, I’m just a lot slower”) and 23 (“I get good and bad days at work”), as they had fit residual greater than ±2.5. These items also misfit in a previous Rasch analysis,^12^ which increases our confidence that there may be problems with these items that warrant their removal. Item 1 is a double-barreled question, as an answer of “no” could mean that the respondent cannot do their job tasks (eg, modified work) or that they can and are not slower (eg, can do normally but have pain). Double-barreled questions are insufficient in terms of content validity. Thus, our Rasch findings that question this item reinforce the need for careful content validity before items are included in a measure and provide justification for removal of the item. Item 23 was designed to indicate the fluctuation of symptoms during day-to-day work but not necessarily for respondents in the recovery phase.^12^ These findings are similar to previous Rasch analyses. Because a single piece of evidence is too preliminary to justify large-scale adoption of a revised measure, this level of conceptual and independent verification is needed. We suggest that these combined concerns justify the removal of these 2 items.

Prior studies have suggested removal of items 8, 13, 20, and 22 to improve the Rasch model fit. However, in the current study, we found no significant deviation from the model fit and retained these items. Because work instability is a complex phenomenon and the questionnaire is already quickly answered due to its yes/no response options, we believe that an overly shortened item may not be sufficiently inclusive of the problems individuals experience. Therefore, where items do function well, we would prefer to retain them. Some differences between Rasch analyses in different studies can be expected due to differences in the health conditions, occupations, and demographics of different study samples. For example, item 22 (“I'm finding any pressure on my hands is a problem”) performed well in our sample in which 25.1% of the injured workers had claims related to the hand and wrist, but may not have been as relevant to patients in a previous study that only included patients with shoulder injuries.^12^ As we see different Rasch analyses being performed on the same instrument, we have come to appreciate that variances in results are to be expected. Therefore, researchers wanting to optimize measurement in their sample should choose a Rasch solution that is most similar to their population. Permanent changes to measures should be considered where there are consistent findings that items do not perform well.

The third item eliminated from the 23-item version to create our 20-item WIS was item 19 (“I get very stiff at work”) since it exhibited nonuniform DIF based on job classification. Usually, nonuniform DIF occurs when the ICC indicates that the participants from different groups with identical ability levels have different probabilities of correctly responding to an item. In the current case, we classified jobs as labor, office work, and mixed. The onset of stiffness could vary between individuals depending on what type of activities they perform. For example, an office worker who works in front of a computer can develop spinal stiffness due to sustained postures, whereas a person with a manual job might have knee and hand stiffness that arises from overuse of muscles and joints. These could be quite different in nature and severity. Because the question is not very specific in terms of the source of stiffness, different jobs could have a different type of response or bias. However, unless this is a stable finding across studies, we are less certain of the recommendation for permanent removal.

Our study identified uniform DIF for item 11 (“I have great difficulty opening some of the doors at work”) for men and women. This did not result in removal of the item because we recognize that differences in response patterns could be due to the biological variations in strength, muscle fibers, etc^33^ because the percentage of strength required for men and women to open a standard door would be different. Uniform DIF for areas of injury was also observed in items 18 (“I'm getting up earlier because of my shoulder/elbow problem”) and 22 (“I'm finding any pressure on my hands is a problem”) in the current study. A similar trend was observed in a previous study by Tang et al,^12^ including uniform DIF for items 18 and 22 between rheumatoid arthritis patients and OA patients. This indicates that patients with different areas of injury in the upper extremity will respond differently to these 2 questions (items 18 and 22). Hence, these 2 items should be separated for different subgroups of patients with various areas of injury.

The transformation of the summed score to an interval-level score in our revised version enables the analysis of within- and between-subject differences such as analysis of variance since interval level scaling is an assumption for mathematical manipulations. Ordinal measures performed by many self-report instruments cannot guarantee that the intervals between different response options are equivalent and, therefore, it would be inappropriate to perform mathematical operations on these scores. We recognize that this is routinely done. However, it violates the assumptions of statistical tests and is a potential source of error.

### Study limitations

A limitation of Rasch analysis is that it can be used to identify problematic items but cannot identify why an item does not perform well. We know that many currently used outcome measures do not have publications that clearly articulate the steps taken to ensure content validity of the items. Issues such as lack of clarity of items, double-barreled questions, and lack of relevance are surprisingly common in currently used outcome measures. Therefore, it is possible that Rasch analysis will indicate the need to delete an item that could be rehabilitated. Some differences exist across different Rasch solutions and only those items that consistently demonstrate measurement problems can be confidently removed^34^ to avoid multiple conflicting versions of outcome measures. However, because shortened measures that remove nonfunctioning items can lessen burden and improve measurement, they should be adopted where sufficient evidence exists.

### Implications

Further research should include cognitive interviewing and qualitative methods to further examine the content validity and clarity of the WIS items to complement statistical approaches and comparison of different Rasch solutions in the same sample to further examine proposed alternate forms, including complementary statistical analyses such as factor analyses and classic psychometric properties (eg, responsiveness). A comparative approach across samples and versions is needed to provide robust evidence on the “best” version of the WIS. At present, researchers should use the version of the WIS that provides the best measurement properties for their study sample.

## Conclusions

In conclusion, we found through Rasch analysis that 3 problematic items of the WIS-23 could be removed to perform a well-functioning interval level scaled WIS-20 in injured workers with upper extremity musculoskeletal conditions. The 20-item WIS-WREUD demonstrated excellent item and person fit, unidimensionality, acceptable person separation index, and local independency.

## Supplier

a. RUMM 2030; RUMM Laboratory Pty Ltd.
