# Supplementary material for: Evaluation of the Structural Validity of the Work Instability Scale Using the Rasch Model
Source: Arch Rehabil Res Clin Transl. 2021 Jan 13;3(1):100103. doi: 10.1016/j.arrct.2021.100103 (PMC7984990; doi:10.1016/j.arrct.2021.100103)
Supplement: Supplemental Table S1 [file mmc1.pdf]

Supplementary Table. The residual correlation between items in 20-item Work Instability Scale

**Item**	I02	I03	I04	I05	I06	I07	I08	I09	I10	I11	I12	I13	I14	I15	I16	I17	I18	I20	I21	I22
I02	1																			
I03	0.14	1.00																		
I04	0.04	-0.07	1.00																	
I05	-0.04	-0.09	-0.05	1.00																
I06	-0.01	-0.08	0.00	0.00	1.00															
I07	-0.01	-0.10	0.05	0.05	0.07	1.00														
I08	0.03	-0.01	0.00	-0.14	-0.01	0.05	1.00													
I09	-0.06	-0.15	-0.07	-0.11	-0.04	-0.20	-0.11	1.00												
I10	-0.13	-0.13	-0.01	-0.09	-0.28	-0.04	-0.13	0.14	1.00											
I11	-0.11	-0.13	-0.04	-0.19	0.02	-0.08	-0.14	-0.02	-0.06	1.00										
I12	0.03	-0.08	0.00	0.00	-0.04	-0.02	-0.08	0.03	0.00	-0.02	1.00									
I13	-0.10	-0.01	-0.01	-0.15	-0.17	-0.08	-0.15	0.27	0.15	0.08	0.01	1.00								
I14	0.11	0.26	-0.06	-0.04	-0.11	-0.15	-0.03	-0.09	-0.09	-0.14	-0.05	-0.10	1.00							
I15	-0.05	-0.04	0.09	-0.07	-0.10	0.11	0.05	-0.10	0.02	-0.02	-0.06	0.02	-0.10	1.00						
I16	-0.08	-0.11	-0.03	-0.01	-0.04	-0.03	0.04	-0.08	-0.03	-0.07	-0.11	-0.15	-0.07	-0.07	1.00					
I17	-0.09	0.06	-0.23	-0.08	0.00	-0.11	-0.03	-0.07	-0.16	-0.09	-0.15	-0.03	0.08	-0.20	-0.04	1.00				
I18	-0.11	-0.17	0.03	-0.06	0.01	-0.05	-0.06	0.01	0.09	-0.02	-0.05	-0.05	-0.10	-0.10	-0.05	-0.05	1.00			
I20	-0.10	-0.01	-0.05	0.00	-0.03	-0.05	-0.07	-0.09	0.06	-0.14	-0.12	0.04	-0.07	0.03	0.02	0.00	-0.02	1.00		
I21	0.01	-0.11	-0.15	-0.05	-0.10	-0.06	-0.07	-0.01	0.03	-0.01	-0.08	-0.09	-0.10	-0.09	0.05	0.06	-0.11	-0.03	1.00	
I22	-0.14	0.01	-0.02	-0.06	-0.08	-0.19	-0.05	-0.09	-0.11	0.10	-0.10	-0.13	-0.01	-0.03	-0.14	-0.01	-0.12	-0.12	-0.09	1.00

Residual correlations between any two items greater than 0.2 above the average residual correlation was taken as an indicator of local dependency
